# Psychopathological Impact and Resilient Scenarios in Inpatient with Schizophrenia Spectrum Disorders Related to Covid Physical Distancing Policies: A Systematic Review

**DOI:** 10.3390/bs11040049

**Published:** 2021-04-13

**Authors:** Pasquale Caponnetto, Alessandra Benenati, Marilena G. Maglia

**Affiliations:** 1 Department of Educational Sciences, University of Catania, 95100 Mascalucia, Italy; abenenati74@gmail.com (A.B.); m.maglia@unict.it (M.G.M.); 2 Center of Excellence for the Acceleration of Harm Reduction (COEHAR), University of Catania, 95100 Mascalucia, Italy; 3 CTA-Villa Chiara Psychiatric Rehabilitation Clinic and Research, 95100 Mascalucia, Italy

**Keywords:** confinement, COVID-19, schizophrenia, social distancing, social isolation, symptomatology, systematic review

## Abstract

The COVID-19 epidemic posed great challenges to the healthcare community. To contain the epidemiological emergency, confinement measures were instituted, affecting the entire population. The lack of social contact, as well as the disruption of daily life, caused the exacerbation of anxiety and depressive symptoms. The present review of the literature aims to investigate what the effects of the pandemic have been on patients with schizophrenia, hypothesizing, an exacerbation of psychotic symptomatology (positive, negative, disorganized symptoms). Between November 2020 and January 2021, 5353 articles were collected and analyzed from the databases of the ResearchGate, Pubmed, and Psycnet websites, subjected to PRISMA methodology. Of these, 11 were evaluated for eligibility, but only three were included in the study because they met all inclusion criteria. The research did not confirm the expected results, showing that any kind of worsening of schizophrenic symptomatology involved the study samples. However, interesting outcomes were highlighted, such as a significant increase in general well-being during the early period of the pandemic, especially by women, or an increase in CPR (C-reactive Protein) levels in the blood, signaling an inflammatory state. Although the systematic review refuted the initial hypothesis, this must be a starting point: the topic is recent and these findings leave ample room for further investigation, particularly in long-term longitudinal research. It is possible that the true response to this disruption of daily life that occurred only during the past year may manifest itself later in time. On the other hand, interesting outcomes have been brought to light that may provide further interesting research insights.

## 1. Introduction

During the first quarter of 2020, the pandemic caused by SARS-CoV-2 spread, quickly sweeping the world. To date, we hear daily about the impact that the Coronavirus has on physical health, from the mildest symptoms to bilateral interstitial pneumonia. However, as well as physical well-being, mental health has also suffered the repercussions of the sudden epidemic. Numerous studies have highlighted the exacerbation of symptoms of anxiety and depression. This is a clear example of the result that the lockdowns, imposed by most States to stem the first wave of spread of the virus, have had on the population.

The analysis of the reaction to these events, in the specifics of the present work, has been oriented towards the response to the pandemic of patients hospitalized in psychiatric institutions, especially those suffering from severe pathologies. Accommodated in nursing homes and rehabilitation centers, patients, in conditions of normality, are not, however, deprived of the social component. The latter, usually, accompanies them in the rehabilitative path, characterized by various activities and the meeting with loved ones. Instead, the new measures adopted to defend all, and, even more, the fragile subjects, have been characterized by the choices of distancing and social isolation.

This literature review aims to investigate the effects of distancing policies, adopted to contain the epidemiological emergency, on patients with schizophrenia in various rehabilitation contexts. The goal is to verify whether modifications of the symptomatologic pattern—characterizing the most serious forms of mental illness—have been detected, focusing in particular on schizophrenia.

Schizophrenia refers to a severe psychopathological framework, psychotic in nature, characterized by the splitting of associative thought processes. This inevitably leads to an imbalance in the normal functioning of the psyche, up to the complete disintegration of mental life. According to the DSM-5, the diagnosis of the disease must include, for a duration of not less than six months, “two or more of the following symptoms [...] one of the symptoms should be 1, 2, or 3:

1. Delusions
2. Hallucinations
3. Disorganized speech
4. Disorganized (or catatonic) behavior
5. Negative symptoms [1]

Physical distancing policies have drastically changed the habits of the entire population. In particular, social interactions have been replaced by prolonged periods of isolation: school activities, as well as work or leisure activities have given way to loneliness and detachment [2].

As the pandemic progressed, researchers documented increases in anxiety and depressive symptomatology, as well as manifestations of intrusive thoughts, sleep disturbances, and substance abuse [2]. For instance, the focus group conducted by Shufang Sun, Danhua Lin, and Don Operario revealed that Chinese students, during the period of mandatory quarantine, experienced psychological distress. It has been manifested through anxious or depressive symptoms, reduced social support, sleep quality, and Internet addiction. In particular, the latter was described as an adaptive strategy for anxiety, insomnia and lack of social interactions [3].

In addition, research suggests that OCD symptomatology has also undergone exacerbations, especially linked to anxiety related to contamination. This can stimulate multiple highly injurious compulsions [4]. Therapeutic intervention, especially during the pandemic, becomes necessary: a worsening of obsessive-compulsive symptomatology is directly proportional to suicidal ideation [5]. OCD symptoms would also appear to have been detected in the animal world, particularly in mice. The interesting research of Gimenez-LIort et al. highlights that the typical digging patterns would seem to have undergone a strong imbalance. The results show that digging patterns undergo a significant increase in new situations (neophobia). This is due to the repetitive behaviors that characterize OCD, useful to reduce distress. On the contrary, in situations of social isolation, the frequency and intensity of digging—a species-specific behavior—is drastically reduced [6]. In summary, novel situations and isolation, even in animals, cause important changes in habitual behavior.

It is precisely in the lack of social interactions that the psychopathological core of the aforementioned malaise would reside. In fact, research suggests that people have the need to be affiliated with others, but if these connections do not exist, they will experience psychological suffering. This need to belong becomes stronger when people experience a stressful situation. The latter is characterized by a lack of a certain future, such as natural disasters, disease, or catastrophes. In these moments, humans seek solace from union with other fellow humans. This reaction is adaptive, as it modulates neuronal and biological responses to stress and, most importantly, coarsens the negative effects of particularly grueling events [2].

The picture of the situation described above is valid for part of the population—in that “most people are resilient and do not succumb to psychopathology” [7]. However, some groups are more vulnerable than others: people who have already contracted the disease, those who have a higher risk of contracting it—the elderly, the immunocompromised or those living in community settings—and people with previous medical and psychiatric conditions. The latter appear to be an ideal target for the manifestation of the worst psychosocial consequences [7].

The pandemic caused by the Coronavirus has had—and continues to have—a strong impact on the entire population, especially on the most fragile categories. Patients fight against the psychological discomforts brought about by confinement and social distancing and simultaneously face additional difficulties related to their pre-existing conditions. In this group, there are also patients suffering from severe mental disorders—including schizophrenia.

According to Ann Shinn and Mark Viron, schizophrenia is associated with cognitive deficits, including dysfunction in executive functions. In addition, individuals with schizophrenia, who are stigmatized by society, usually have lower levels of education, lower self-control, lower self-care than average and inadequate insight. These elements influence the greater difficulty that these patients manifest to find correct information about Coronavirus and to be able to translate the procedures of prevention of contagion in behaviors [8].

In this regard, risk perception and adherence to protective measures in individuals diagnosed with schizophrenia should be of particular concern to their caregivers. The review by Brown et al. focused on the impact of successive epidemics throughout history (including SARS, MERS, Ebola, and swine flu) on psychosis. The research found that patients diagnosed with schizophrenia are less likely to be vaccinated and isolated. In addition, there is a positive correlation between psychotic symptoms and poor adherence with protective measures. On the other hand, patients with schizophrenia are well aware of epidemic risk perception (particularly in studies concerning SARS and swine flu) [9].

Specifically, patients hospitalized in nursing homes would be more susceptible to SARS-CoV-2 infection, as they share common spaces, such as bathrooms or dining rooms. In addition, psychiatric patients usually participate in group activities, which increase interpersonal contact [10].

For this reason, policies have been instituted to contain the spread of the virus, even within psychiatric institutions. For instance, patients are asked not to go outside their wards, visits from families have been cancelled and no cell phones or other electronic devices are allowed. Moreover, no information on the Internet is made available to individuals [11]. However, these measures of social isolation reduce and collapse the weak social networks built by patients [8].

From an organic perspective, people suffering from schizophrenia would also appear to be more vulnerable to the adverse consequences of the new illness. More than 70% of patients have experienced at least one other clinical condition, such as type-2 diabetes, chronic lung disease, or heart disease. This would increase the mortality rate caused by COVID-19 in individuals with schizophrenia [12].

Moreover, even the choice of neuroleptic therapy may expose patients to more predisposing conditions towards Coronavirus contraction. For example, the use of clozapine—a second generation antipsychotic particularly effective in the treatment of refractory schizophrenia—would not be recommended for use because of agranulocytosis, a dangerous side effect [12].

The massive modifications of the social network present a surreal scenario to which it is difficult to get used. For those who suffer from psychotic disorders, this new reality may exacerbate feelings of perplexity, anxiety, and paranoia. Furthermore, the current situation may be assimilated into the delusional ideational contents, typical of the patient diagnosed with schizophrenia [8].

On the other hand, research against Coronavirus does not stop. The latest theoretical findings show the existence of drugs that have a protective effect on COVID-19. They use a mechanism of action based on the inhibition of acid sphingomyelinase (FIASMAs). This awareness not only acts as a protective factor but could reduce the anxiety of contagion (presumably not only in patients with schizophrenia) [13].

In light of the above, the following systematic review explores the consequences of Covid-19 confinement policies on patients diagnosed with schizophrenia or other severe mental illnesses. Specifically, it investigates possible exacerbation or modification of positive, negative, or disorganized symptomatology, typical of the schizophrenic picture.

## 2. Materials and Methods

### 2.1. Objective of the Research

The objective of this research is to verify what the effects of physical distancing policies—approved as a result of the COVID-19 epidemiological emergency—have been on patients in rehabilitation settings suffering from severe mental illnesses, particularly schizophrenia.

### 2.2. Literature Search and Selection

The literature review was performed following the 2009 PRISMA guidelines for Systematic Reviews. The literature search was conducted during the time period of November 2020 to January 2021, in the databases of ResearchGate, Pubmed, and Psycnet websites. The keywords used were “COVID-19 and Schizophrenia”, “COVID-19 and mental illness”, and “COVID-19 and social isolation”.

### 2.3. Eligibility Criteria

For the study, all articles written in English with no time restrictions regarding publication date that met the following criteria were considered:

1. Participants: patients with schizophrenia.
2. Intervention: effects of COVID-19 physical distancing policies on symptomatology.
3. Comparison: symptomology before the COVID-19 pandemic.
4. Outcome: exacerbation or decrease of positive, negative, and disorganized symptomatology.
5. Study design: experimental studies, longitudinal studies.

### 2.4. Data Extraction

Data extraction highlighted, for each article, author, year, article title, country in which the research took place, type of study, sample, instruments used and results.

### 2.5. Risk of Bias Assessment

Regarding the calculation of risk of bias, the selected studies did not report randomization procedures; therefore, the “Cochrane risk-of-bias tool for randomized trials, version 2 (RoB 2)” could not be used.

## 3. Results

**Figure 1:**
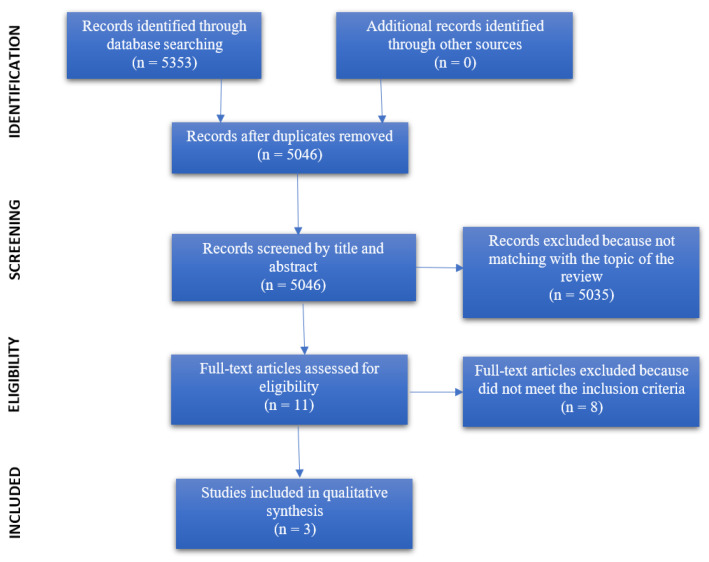
PRISMA 2009 flow diagram.

The articles resulting from the search phase in the previously listed databases resulted in a total of 5353 articles. No other noteworthy articles from other sources were identified. After the first skimming phase, with the elimination of 307 duplicates, the remaining 5046 articles were analyzed by reading the title and abstract. Among these, as many as 5035 papers were excluded because they did not simultaneously address Coronavirus and patients with schizophrenia. The 11 remaining articles were assessed as eligible and were read entirely. Of these, eight articles were excluded because they did not meet the inclusion criteria (in particular, schizophrenia symptomatology was not investigated) and one article described a protocol that is still being tested. Finally, the included studies in the qualitative synthesis are three. The aforementioned description is summarized in the flowchart in Figure 1, while the data extraction of these studies can be viewed in Table 1.

In the study by Pinkham et al. [14] is made a comparison between pre and post pandemic symptoms. The aim is to examine whether there has been an exacerbation of the conditions of patients with serious mental illnesses.

The study sample included 148 individuals, 92 of whom had schizophrenia.

Participants first underwent a baseline examination during which diagnosis and severity of symptomatology were confirmed; instruments used included PANSS, MADRS, YMRS, SUMD.

Pre-pandemic data were collected using an Ecological Momentary Assessment (EMA) during two different studies; the questionnaire was administered three times a day for 30 days (study 1—December 2018) or 10 days (study 2—July 2019). The instrument included items related to daily activities, mood, and psychotic symptoms (hearing voices or having paranoid thoughts). Individuals were also asked to assess sleep quality and, only on the last questionnaire of the day, substances used and total level of well-being.

Data collection for the COVID-19 period was carried out via a telephone survey, during which EMA items were administered again.

What comes from this longitudinal study is the absence of significant changes in mood or psychotic symptoms in the two periods considered. This could be an indication of a good coping response by the patients.

The study by Jun Ma et al. [15] investigates the impact that social distance caused by COVID-19 epidemiological containment policies has on patients with schizophrenia. The emphasis is on common inflammatory indexes and psychological characteristics.

The study sample included 30 patients suffering from schizophrenia from the Wuhan Mental Health Centre, who were subjected to social isolation after having contact with a COVID-19-positive patient. There was also established a control group of 30 patients with schizophrenia, likewise from the Wuhan Mental Health Centre, who were not subjected to isolation.

The instruments used for data collection were: CPSS, PANSS, HAMD, HAMA, and PSQI. Stress, psychotic symptoms (positive and negative), depressive symptoms, anxiety, and sleep quality were investigated.

Even in this study, there were no significant differences in changes in schizophrenic symptomatology, either between the experimental group and the control group, or within the experimental group itself. The aforementioned instruments were administered before and after the isolation period.

In the study by Hannah L. Quittkat et al. [16] the focus was on the psychological consequences COVID-19 had on all mental disorders.

The sample, consisting of 2233 individuals, included only six patients with schizophrenia or other psychotic disorders.

Participants were submitted to an online survey which investigated current psychological conditions and asked participants to respond, retrospectively, thinking about their own conditions in the November 2019 timeframe. The questionnaire included items from several instruments, including FKS, CAHSA, DASS-D, and PHQ.

As far as individuals with schizophrenia are concerned, no differences in psychotic symptoms were found in the two time periods considered.

### 3.1. Schizophrenic Symptomatology

From the three studies considered, it was found that there was no exacerbation or decrease in positive, negative, or disorganized symptoms assessed by PANSS or CAHSA.

### 3.2. Other Results

Although the research did not confirm the expected results, there were highlighted interesting outcomes.

The study by Pinkham et al. [14], compared to the pre-pandemic period, reports an increase in the number of substances taken and, unexpectedly, a significant increase in overall well-being during the early period of the pandemic. The latter was experienced especially by women (the authors suggest that this gender difference could be an additional research focus). The authors add that, presumably, this finding may stem from spending less time alone before the pandemic outbreak, which is associated with resilience.

The case-control study by Jun Ma et al. [15], meanwhile, shows that patients who were subjected to isolation had higher levels of stress, anxiety, depression, and worse sleep quality than the control group that was not in isolation. In addition, there was also an increase in CPR (C-reactive protein) levels in the blood, which signals an inflammatory state: Social isolation is a special form of inflammation [15].

Regarding the study by Hannah L. Quittkat et al. [16], the authors point out that the study has limitations related to the small sample, consisting of only six individuals with schizophrenia. However, also in this case were found higher levels of stress and changes in behaviors related to hygiene (all groups take more time to wash their hands), shopping (reduced weekly frequency of shopping) and reduced social contacts.

## 4. Discussion

Research has shown that, to date, COVID-19 epidemiologic emergency distancing policies have not made any significant symptomatic changes for patients with schizophrenia or other serious mental illnesses. What emerges at the moment is a demonstration of resilience on the part of patients with schizophrenia diagnosis, as evidenced by the research of Pinkham et al. [14]. Another explanation may lie in the very definition of the disorder. Schizophrenia is a heterogeneous disorder, encompassing several clinical manifestations. Consider, for example, ideas of persecution or, more simply, delusions. In the first case, social isolation-generalized by the pandemic-in a psychotic patient may reassure him or her because of fear of persecution. Or, in the second case, social isolation may be absorbed within the patient’s delusion. Considering the above, the consequences of the pandemic in individuals diagnosed with schizophrenia are variable and subjective. This depends on the symptomatology experienced by each patient. In any case, the phenomenon taken into consideration is very recent: The bibliography is still being updated. Moreover, as the authors of the articles also suggest, subsequent longitudinal studies will be essential to verify, in the long-term, the consequences of the pandemic period. In addition, it must also be taken into account the reference sample that, in the case of the research of Hannah L. Quittkat et al. [16], represents only 0.27% of the total number of participants (six subjects out of 2233).

However, interesting results emerged from the researches: social distancing affected sleep quality, stress, anxiety and depression, exacerbating them. Furthermore, at a physiological level, it has been shown how social isolation can stimulate the production of CPR, raising inflammatory indices up to a statistically significant level compared to a normal condition. The perception of well-being experienced by patients suffering from schizophrenia during the early stages of the pandemic is also of particular interest. The latter, according to the authors (Pinkham et al.) [14], could be due to an increase in social contacts before the pandemic.

## 5. Conclusions

This systematic review of the literature sought to highlight the immediate consequences of the global COVID-19 epidemic on individuals with schizophrenia or severe mental illness. What we wanted to investigate is, in particular, a possible exacerbation, or modification, in schizophrenic symptomatology, concerning positive, negative, and disorganized symptoms. This hypothesis was refuted by the research under analysis, which showed that no significant change involved these symptoms taken into consideration. However, this should not be a point of arrival, rather a point of departure: The topic is recent and these results leave ample room for further investigation, particularly in long-term longitudinal research. It is possible that the true response to this upheaval in daily life that occurred only during the past year may manifest itself later in time.

The research also highlighted interesting implications as a result of social distancing, such as increased production of C-reactive protein or changes in habitual behaviors (going shopping or washing hands).

We hope, therefore, that research can continue in this direction, to nourish and increase this still uncultivated field in order to be able to benefit at the clinical level, in patient care.

## Figures and Tables

**Table 1:** Articles included in the review. Legend: PANSS = The Positive and Negative Syndrome Scale; MADRS = Montgomery–Asberg Depression-Rating Scale; YMRS = Young Mania Rating Scale; SUMD = Scale to Assess Unawareness of Mental Disorder; EMA = Ecological Momentary Assessment; CPSS = Chinese Perceived Stress Scale; HAMD = Hamilton Depression Scale; HAMA = Hamilton Anxiety Scale; PSQI = Pittsburgh sleep quality index; FKS = Body Dysmorphic Symptoms Inventory; CAHSA = Continuum of Auditory Hallucinations—State Assessment; DASS-D = Depression Anxiety Stress Scales—Depression Subscale; EDE-Q = Eating Disorder Examination-Questionnaire; PHQ = Patient Health Questionnaire—Panic Module and Stress Subscale; PSWQ-d = Penn State Worry Questionnaire; SIAS = Social Interaction Anxiety Scale; SPS = Social Phobia Scale, WI = Whitely Index; Y-BOCS = Yale–Brown Obsessive Compulsive Scale—Symptom Checklist.

**Authors**	**Year**	**Title**	**Country**	**Study Design**	**Total Sample**	**Instruments**	**Results**
Amy E. Pinkham, Robert A. Ackerman, Colin A. Depp, Philip D. Harvey, Raeanne C. Moore	2020	A Longitudinal Investigation of the Effects of the COVID-19 Pandemic on the Mental Health of Individuals with Pre-existing Severe Mental Illnesses	USA	Longitudinal study	148 individuals suffering from serious mental illness; 92 individuals suffering from schizophrenia	PANSS, MADRS, YMRS, SUMD, EMA questionnaire	Contrary to expectations, there were no significant changes in positive, negative, or disorganized symptomatology compared with the pre-pandemic situation. In contrast, there is a significant increase in well-being in the early pandemic period.
Jun Ma, Tingting Hua, Kuan Zeng, Baoliang Zhong, Gang Wang, Xuebing Liu	2020	Influence of social isolation caused by coronavirus disease 2019 (COVID-19) on the psychological characteristics of hospitalized schizophrenia patients: a case-control study	China	Case-control study	30 patients with schizophrenia subjected to isolation as the experimental group; 30 patients with schizophrenia not subjected to isolation as the control group	CPSS, PANSS, HAMD, HAMA, PSQI	The results of the study show that patients in isolation experience higher levels of stress, anxiety, and depressive symptomatology, compared to patients not in isolation. However, PANSS scale scores between the two groups are not significantly different, meaning that no relevant changes in schizophrenic symptomatology are detected.
**Authors**	**Year**	**Title**	**Country**	**Study Design**	**Total Sample**	**Instruments**	**Results**
Hannah L. Quittkat, Rainer Düsing, Friederike-Johanna Holtmann, Ulrike Buhlmann, Jennifer Svaldi, Sija Vocks	2020	Perceived Impact of Covid-19 Across Different Mental Disorders: A Study on Disorder-Specific Symptoms, Psychosocial Stress and Behavior	Germany	Quantitative research (questionnaire)	2233 individuals diagnosed with mental illness. 6 individuals having schizophrenia and other psychotic disorders.	FKS, CAHSA, DASS-D, EDE-Q, PHQ, PSWQ-d, SIAS, SPS, WI, Y-BOCS	Psychotic symptoms, compared to the pre-pandemic situation, do not appear to have undergone any modification. However, this could be due to the very small sample (only 6 individuals).

## Data Availability

Not applicable.

## References

[1] Kring A.M., Johnson S.L., Davison G.C., Neale J.M. (2017). Schzofrenia. Psicologia Clinica.

[2] Marmarosh C.L., Forsyth D.R., Strauss B., Burlingame G.M. (2020). The Psychology of the COVID-19 Pandemic: A Group-Level Perspective. Group Dyn. Theory Res. Pract..

[3] Sun S., Lin D., Operario D. (2020). Need for a Population Health Approach to Understand and Address Psychosocial Consequences of COVID-19. Psychol. Trauma Theory Res. Pract. Policy.

[4] Fineberg N.A., Van Ameringen M., Drummond L., Hollander E., Stein D.J., Geller D. (2020). How to manage obsessive-compulsive disorder (OCD) under COVID-19: A clinician’s guide from the International College of Obsessive Compulsive Spectrum Disorders (ICOCS) and the Obsessive-Compulsive and Related Disorders Research Network (OCRN) of the European College of Neuropsychopharmacology. Compr. Psychiatry.

[5] Farhan R., Llopis P. (2020). Psychiatric and neuropsychiatric syndromes and COVID-19. Lancet Psychiatry.

[6] Gimenez-LIort L., Alveal-Mellado D. (2021). Digging Signatures in 13-Month-Old 3xTg-AD Mice of Alzheimer’s Disease and Its Disruption by Isolation Despite Social Life Since They Were Born. Front. Behav. Neurosci..

[7] Pfefferbaum B., North C.S. (2020). Mental Health and the Covid-19 Pandemic. N. Engl. J. Med..

[8] Shinn A.K., Viron M. (2020). Perspectives on the COVID-19 pandemic and individuals with serious mental illness. J. Clin. Psychiatry.

[9] Brown E., Gray R., Monaco S.L., O’Donoghue B., Nelson B., Thompson A. (2020). The potential impact of COVID-19 on psychosis: A rapid review of contemporary epidemic and pandemic research. Schizophr. Res..

[10] Xiang Y.T., Zhao Y.J., Liu Z.H., Li X.H., Zhao N., Cheung T., Ng C.H. (2020). The COVID-19 outbreak and psychiatric hospitals in China: Managing challenges through mental health service reform. Int. J. Biol. Sci..

[11] Li S., Zhang Y. (2020). Mental healthcare for psychiatric inpatients during the COVID-19 epidemic. Gen. Psychiatry.

[12] Khosoravi M. (2020). COVID-19 Pandemic: What are the Risks and Challenges for Schizophrenia?. Clin. Schizophr. Relat. Psychoses.

[13] Le Corre P., Loas G. (2021). Repurposing functional inhibitors of acid sphingomyelinase (fiasmas): An opportunity against SARS-CoV-2 infection?. Clin. Pharm. Ther..

[14] Pinkham A.E., Ackerman R.A., Depp C.A., Harvey P.D., Moore R.C. (2020). A Longitudinal Investigation of the Effects of the COVID-19 Pandemic on the Mental Health of Individuals with Pre-existing Severe Mental Illnesses. Psychiatry Res..

[15] Ma J., Hua T., Zeng K., Zhong B., Wang G., Liu X. (2020). Influence of social isolation caused by coronavirus disease 2019 (COVID-19) on the psychological characteristics of hospitalized schizophrenia patients: A case-control study. Transl. Psychiatry.

[16] Quittkat H.L., Düsing R., Holtmann F.J., Buhlmann U., Svaldi J., Vocks S. (2020). Perceived Impact of Covid-19 Across Different Mental Disorders: A Study on Disorder-Specific Symptoms, Psychosocial Stress and Behavior. Front. Psychol..

